# Acceptability of the MySmile Mobile Health Application Among Malaysian Adolescents: A Qualitative Study Using the Theoretical Framework of Acceptability

**DOI:** 10.7759/cureus.90267

**Published:** 2025-08-17

**Authors:** Muhammad Hamidie Saari, Jamaludin Marhazlinda, Unaizah Hanum Obaidellah, Nazirah Ab Mumin, Zamros Yuzadi Mohd Yusof

**Affiliations:** 1 Department of Community Oral Health and Clinical Prevention, Faculty of Dentistry, Universiti Malaya, Kuala Lumpur, MYS; 2 Department of Artificial Intelligence, Faculty of Computer Science and Information Technology, Universiti Malaya, Kuala Lumpur, MYS; 3 Department of Periodontology and Community Oral Health, Faculty of Dentistry, Universiti Sains Islam, Kuala Lumpur, MYS

**Keywords:** children and adolescents, degree of gingivitis, flossing technique, mobile health, mobile health application, oral health education (ohe), qualitative studies, school oral health promotion, self-efficacy, toothbrushing

## Abstract

Background

Oral health among adolescents remains a public health concern in Malaysia, with persistent challenges in achieving behavioural change through traditional education methods. Given the widespread use of smartphones among adolescents, mobile health (mHealth) applications offer a promising avenue for health promotion.

Objective

This study aimed to explore the acceptability of the MySmile mobile application (app), designed to improve oral health knowledge and behaviours among secondary school students in Malaysia, using the Theoretical Framework of Acceptability (TFA).

Methods

This study employed a qualitative approach that involved five focus group discussions (FGDs) with 23 students from two intervention schools that participated in a 12-week MySmile app intervention. Participants were purposively selected based on their gender, school locality, and frequency of app usage. Discussions were guided by a semi-structured topic guide aligned with the seven TFA domains: affective attitude, burden, ethicality, intervention coherence, opportunity cost, perceived effectiveness, and self-efficacy. Data were transcribed verbatim and analysed using the Framework Method.

Results

The app was perceived as highly acceptable across all TFA domains. Students described it as fun, engaging, and easy to use, with gamified features that enhanced learning. The perceived burden was low, and ethicality was reinforced by the app’s culturally appropriate content and privacy features. Participants demonstrated a clear understanding of the app’s purpose and reported no significant opportunity cost. Perceived effectiveness was strong, with students reporting improved brushing habits, reduced sugar intake, and sharing of health messages with family. The app also promoted self-efficacy, with students expressing confidence in applying its recommendations independently.

Conclusions

The MySmile app was well accepted among Malaysian adolescents and appears to be a feasible tool for school-based oral health promotion. These findings support the integration of mHealth strategies in adolescent health programmes and highlight the importance of user-centred, theory-driven intervention design.

## Introduction

Oral health is a fundamental aspect of general health, yet it remains one of the most neglected areas among adolescents, particularly in low- and middle-income countries [1]. In Malaysia, the oral health status of school-aged children continues to raise concern, with high prevalence rates of dental caries and poor oral hygiene reported in national surveys [2,3]. These issues persist despite the long-standing implementation of school-based oral health programmes which largely rely on classroom lectures and printed materials. Such traditional approaches often lack engagement and fail to produce long-term behavioural change [4].

The widespread use of smartphones among Malaysian adolescents presents a timely opportunity to explore digital interventions in oral health promotion. According to the Malaysian Communications and Multimedia Commission, over 90% of adolescents own or regularly use a smartphone [5]. Mobile health (mHealth) applications, which deliver oral health education (OHE) through digital devices, have gained traction as scalable, cost-effective tools that can complement existing OHE efforts [6]. Emerging evidence suggests that mHealth tools, when properly designed, can positively influence oral health knowledge, attitudes, and behaviours among adolescents [7].

To capitalise on this potential, the MySmile app was locally developed as part of a broader intervention strategy targeting oral health improvement among Malaysian secondary school students [8]. The app offers four main features that work together to support learning, behaviour change and daily engagement. First, the Knowledge Hub includes seven educational topics covering essential areas such as brushing and flossing, dental caries, gum disease, malocclusion, teeth whitening, bad breath, and how diet affects oral health. To make learning more interactive, it also includes two fun games: Game of Streaks, which rewards users for consecutive correct answers, and The Teeth Quiz, a fast-paced challenge with points added or deducted based on accuracy. A leaderboard motivates users by ranking their performance and encouraging healthy competition. Second, the Track Yourself feature lets users log their toothbrushing routines and sugar intake. These logs are shown in simple 14-day graphs to help students see their progress and build consistent habits. Third, the Get Help section provides quick access to commonly asked questions on oral health. It also helps users find nearby dental clinics, making it easier for them to seek professional care when needed. Finally, the app sends Friendly Reminders to encourage users to brush their teeth and reduce sugar consumption daily.

The MySmile app is designed for Android smartphones and does not collect personal data. It focuses on creating a safe, easy-to-use, and motivating experience for teenagers. While its impact on oral health knowledge, habits, and clinical outcomes has been studied, how well it is received by adolescents is still an area that needs further exploration. 

Understanding acceptability is critical for the successful implementation and sustainability of digital health interventions. Sekhon et al define acceptability as a multifaceted construct that reflects the cognitive and emotional responses of individuals to an intervention [9]. The Theoretical Framework of Acceptability (TFA) was developed to provide a structured approach to assess this construct across seven domains: i) Affective attitude refers to how individuals feel about using the intervention, ii) Burden describes the amount of effort required to participate in the intervention, iii) Ethicality relates to how well the intervention aligns with the individual’s values, iv) Intervention coherence reflects how well the individual understands the purpose and function of the intervention, v) Opportunity cost considers what the individual might have to give up to engage with the intervention, vi) Perceived effectiveness refers to the extent to which the individual believes the intervention will achieve its intended goals, and vii) Self-efficacy relates to the individual’s confidence in their ability to use and engage with the intervention as intended. Compared to other health behaviour frameworks such as COM-B [10] or the Health Belief Model [11], the TFA is specifically designed to evaluate users’ subjective experiences during or after interacting with an intervention, making it highly suitable for exploring mHealth app acceptability in real-world adolescent settings [9].

The objective of this study was to explore the acceptability of the MySmile app among Malaysian secondary school children using the TFA as a guiding framework. The findings can be used to inform the refinement of the app and provide insights for the integration of the MySmile app into national oral health promotion policies targeting adolescents.

## Materials and methods

The study was conducted in accordance with the Consolidated Criteria for Reporting Qualitative Research (COREQ) checklist and adhered to established standards of rigour in qualitative study [12].

Study design

This study employed a qualitative approach to gain insights into how individuals interpret their experiences and the meaning a phenomenon holds for them [13]. It was part of a larger study assessing the effectiveness of the app in a cluster randomised controlled trial. In this qualitative study, focus group discussions (FGD) were used to allow secondary school students to give their opinions on the effectiveness of the MySmile apps during their interaction and communication with each other in a group setting. With FGD, ideas can be generated from all the participants to be further discussed and explored. Furthermore, through this meeting, it also allows the researcher to witness their non-verbal behaviours and gain further insights into their views of the app and its utility. Therefore, FGD is a suitable method for evaluating the effectiveness of a mobile app and identifying the needs of the target group for further improvement of the app if indicated.

Development of FGD questions and training of facilitators

A semi-structured topic guide was developed based on the seven constructs of the TFA: affective attitude, burden, ethicality, intervention coherence, opportunity cost, perceived effectiveness, and self-efficacy [9]. It was checked and validated by experts in the dental public health discipline to ensure the topic guide addressed the study objectives before it was reviewed by three qualitative research experts. A trial FGD was conducted among six 16-year-old students. It was facilitated by an expert-trained researcher (MHS) to test the draft semi-structured questions. The session was also aimed at providing experience to the moderator and to assess the FGD feasibility under field conditions. Based on the trial, the topic guide was modified and later finalised. The principal researcher is a male doctoral candidate in Dental Public Health with prior clinical experience and training in qualitative research. To minimise bias, he was not affiliated with the participating schools and had no prior relationship with any of the students. While his background in oral health may have shaped the formulation of the probing questions, conscious efforts were made to remain neutral during data collection.

Sampling and recruitment

The study was conducted at two selected government-funded, coeducational secondary schools located in urban and rural areas of the Kinta District, Perak, Malaysia. These schools had previously participated in the MySmile app intervention arm, where students were instructed to use the app on a daily basis for four weeks, and they were encouraged to continue using it after that. The intervention lasted over 12 weeks. A list of students who participated in the MySmile app intervention (n = 167) from the two schools was obtained from the school teachers. Participants were purposively selected after the intervention had been completed from the 167 participants. Maximum variation sampling was employed to ensure heterogeneity in gender, ethnicity, and school locality (urban and rural) [14]. For this purpose, first, we asked the teachers to list the students into three categories, i.e., those who used the app daily, most days of the week, and occasionally (a few times a week). Next, we purposely selected students from each of the categories into a focus group with variations in gender, age, ethnicity, and school locations. The inclusion criteria were: (1) students aged 15 to 16 years, (2) some prior experience using the MySmile app during the intervention period, and (3) ability to communicate fluently in either Malay or English. Students with known cognitive impairments or learning disabilities were excluded from participation.

Data collection, analysis and rigour of study

Data collection took place after the 12-week intervention in December 2024. The FGD was conducted face-to-face in a designated room in each school. This provided a familiar and safe environment for the participants. Each FGD included four to six participants. Before the FGD began, the participants filled in their background data in a standardised form. Next, the facilitator (MHS) introduced himself, followed by the participants to break the ice. The participants were informed that they were allowed to ask questions during the session, and the FGD would be recorded using an audio recorder with their consent. After that, the facilitator asked the first open-ended question and the participants were allowed to discuss actively and give opinions until no new points were raised. The discussion was primarily conducted in the Malay language and occasionally in English, where appropriate. Next, the facilitator would continue by asking the second question, and the process was repeated until all the questions were asked by the facilitator. Each session lasted for about 40 to 60 minutes. The FGD was assisted by a note-taker who recorded the time and essential points from the discussion. Four FGDs were initially planned to represent each key subgroup: urban Form Three (15 years old), rural Form Three (15 years old), urban Form Four (16 years old), and rural Form Four (16 years old). An additional group (urban Form Four) was recruited sequentially following this stratification to confirm data saturation, after which data collection was stopped. 

Data were analysed using the framework method [15]. First, the recorded voice data were transcribed verbatim in the Malay language using Notta ai software, and translated into English by a team of two expert translators, i.e., an English language lecturer who was fluent in the Malay language, and a professional translator. The translation was done via literal translation sticking closely to the source of text wording. Transcripts were anonymised, and unique identifiers were assigned to each participant. Next, the researcher (MHS) repeatedly read the transcripts to become familiar with the raw data. This was followed by the coding process conducted by MHS, where initial themes were generated by means of a free coding technique aided by Atlas.ti software. Following this, a framework was created, and the coded data were indexed into it. Finally, the data were summarised and interpreted according to the TFA domains. To improve accuracy, two other researchers (NSC and MNS) were given the coding and the transcripts for checking. Discrepancies in the interpretations were resolved through peer debriefing and discussion.

In this study, several steps were taken to ensure the trustworthiness of the qualitative data. The study followed recognised strategies to enhance data credibility, dependability, and confirmability [16]. These included conducting a pilot test to test the relevance, clarity, and appropriateness of the topic guide. To build rapport and promote openness, the moderator spent several minutes interacting informally with the participants before starting the FGD. This helped reduce participant anxiety and encouraged more natural and honest exchanges during the discussion. Finally, data triangulation was performed by inviting researchers in the same institution (ZYMY) and outside (NA) to check the coded transcripts with the finalised themes. This reduced individual bias and ensured the findings accurately reflected the participants' perspectives.

Ethical considerations

This study was approved by the Medical Research and Ethics Committee (MREC), Ministry of Health Malaysia (NMRR-24-02037-CA0) and Medical Ethics Committee, Faculty of Dentistry, Universiti Malaya (DF CO2405/0014 P). Permissions to conduct the study were obtained from the Ministry of Education, the State Education Departments, and participating schools. Written informed consent was obtained from participants and parents or legal guardians. Confidentiality and anonymity were strictly maintained throughout the research.

## Results

A total of 23 students from the two intervention schools participated in five FGDs, i.e. three groups of five participants each group, and two groups of four participants each group. Table 1 summarises the demographic characteristics of the 23 students who participated in the FGD. The sample included a balanced distribution in terms of gender, age, and academic level, with nearly equal representation from Form Three and Form Four. Participants were also selected to reflect diverse school locations (urban and rural) and varied frequency of app usage, ensuring a wide range of perspectives were captured.

**Table 1:** Profiles of Participants Involved in the Focus Group Discussion.

Variable	Total, n (%)
Gender	
Male	13 (56.5)
Female	10 (43.5)
Age	
15 years old	12 (52.2)
16 years old	11 (47.8)
School location	
Urban	11 (47.8)
Rural	12 (52.2)
Ethnicity	
Malay	21 (91.3)
Indian	2 (8.7)
Academic Performance	
Form Three	12 (52.2)
Form Four	11 (47.8)
Total	23 (100)

**Figure 1:**
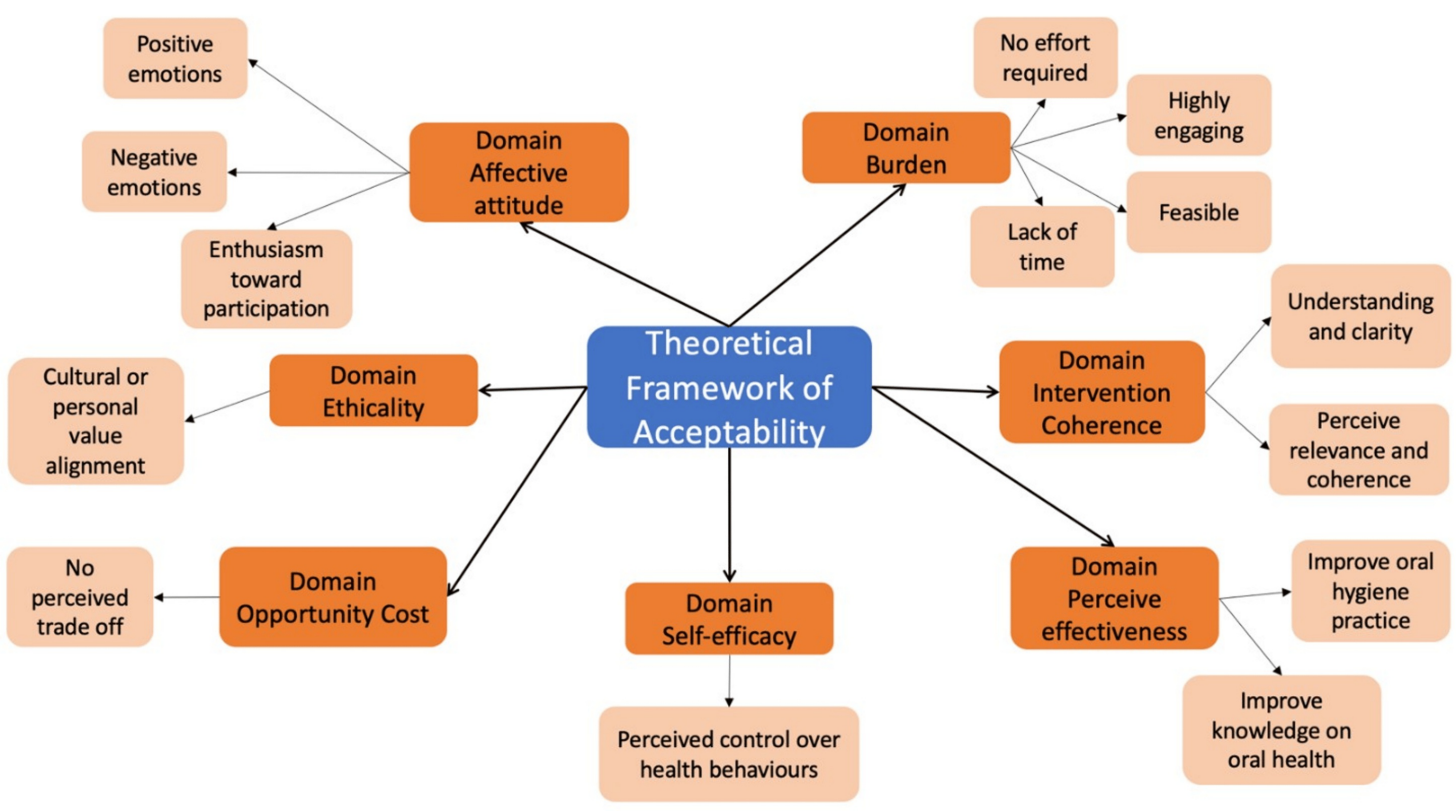
The Themes That Emerged From the Theoretical Framework of Acceptability Domains

Data analysis revealed 14 themes mapped to the seven TFA domains: affective attitude, burden, ethicality, intervention coherence, opportunity cost, perceived effectiveness, and self-efficacy. Figure 1 shows an overview of the themes that emerged from the FGDs.

Affective attitude

Affective attitude deals with how users feel about the app. There were three themes emerged under this domain, i.e., positive emotions, negative emotions, and enthusiasm toward participation.

Positive Emotions

Overall, participants reported a positive experience using the MySmile app. They found the app's content to be relevant to their oral health needs, and appreciated that it was easy to incorporate it into their daily routines, such as during morning or bedtime toothbrushing. The participants felt they experienced a high level of comfort with regular use of the app. 

“On the first day, I really explored the app. I clicked on various features just to explore it. For me, this app is very relevant for teenagers like me to understand dental knowledge in more depth.” (Participant 1, Male, Urban school)

“The app is easy to use, especially if we don’t know much about dental care. For example, how to properly clean your teeth, use floss, and all that. Then, there are games in the app, so it’s helpful for beginners like me.” (Participant 4, Male, Urban school)

Some students proposed that the app be broadened to include iOS users, rather than Android users only. They appreciated the features in the app which facilitated the enhancement of knowledge.

“I hope this app is available on iOS and not limited to Android users only. It’s difficult for me because I like the knowledge part, but I always have to borrow my mom’s phone.” (Participant 11, Female, Urban school)

Negative Emotions

While the majority had positive experiences, a few students expressed their frustration or dissatisfaction with certain features of the app, which generated negative emotional responses. Some users found parts of the app confusing or difficult to locate, which led to feelings of frustration. For example, a few participants were confused about how to access the Habit tracker mode, leading to delays in engaging the app's core function.

"I felt there was a bit of confusion earlier. The app doctor asked us to tick something in the Habit tracker mode, but I was confused about where that part was. I spent a whole week looking for it, but I couldn’t find it." (Participant 14, Female, Rural school)

Several users admitted that the app could be slightly annoying due to the Reminder function, especially during busy times. Although they didn’t fully reject the reminder, the timing of reminders could be intrusive sometimes.

"Sometimes I feel a bit annoyed seeing this all the time. It all feels kind of troublesome... but if I am really busy, I can just slide the notification away. So, it's not too much of a hassle." (Participant 15, Male, Rural school)

A few users noted that they had some difficulties in correcting or editing entries if they had forgotten to log a behaviour that occurred on the previous day into the app. This had raised concerns about the accuracy and fairness of the entries.

“One part that I find troublesome is that we can’t edit entries in the habit tracker. If we forget to tick yesterday’s entry, we can’t tick that column anymore, even though I did brush my teeth yesterday.” (Participant 10, Female, Rural school)

Enthusiasm Toward Participation

Some participants showed enthusiasm for using the app. They described the experience as enjoyable and were motivated by how the app made OHE accessible and personally relevant. For example, several participants shared their excitement about the initial set of questions presented before logging in, which helped determine their oral health status. They found this feature not only informative but also empowering.

“For me, this app is very good. When you first open the app, before logging in, there’s a set of questions to help determine your profile status. That’s very helpful because it tells us about our oral health status. Usually, you’d have to visit a dentist to find out your oral health status, but just by answering these questions, we can already know our status. I was amazed for a moment.” (Participant 8, Female, Rural school)

They also provided suggestions for improving the app. Some of the suggestions they offered were to enable a bilingual version of the app, to add more quizzes and games and to add a reward system to promote long-lasting engagement.

“I feel very excited to use this app, and I suggest that the MySmile app can create a bilingual version so that it’s easier for us to understand the content because not all speaks and understands English fluently.” (Participant 7, Female, Rural school)

“This app can add a "wow factor" on top of the current features as we discussed earlier, by creating more games and a reward system to attract more people to download this app and achieve better engagement in the long run.” (Participant 1, Male, Urban school)

Burden

In this domain, burden relates to the perceived effort required to use the MySmile app. Four themes emerged, i.e., no effort required, highly engaging, feasible and lack of time.

No Effort Required

Most participants felt that this app was not a burden for them as it didn’t require much effort to use, and it didn't take much of their time. Some mentioned that the app was more about helping users to change behaviours and adopt healthy habits because it had useful features like Reminders and the Habit tracker. These features were said to be very useful and helpful to remind them what to do every day.

“For me, this app is not burdensome at all because using it takes a short amount of time, and the storage it uses is not too much.” (Participant 20, Male, Urban school)

“It’s not troublesome at all because it’s easy to use with little effort. You just have to tick things off in the habit tracker, and since I’m always on my phone, it’s not difficult to do at all.” (Participant 16, Male, Urban school)

“The MySmile app can act as a reminder, meaning that if they forget, the MySmile app can remind them to brush their teeth, monitor sugar intake, and so on. It also has a way to track your brushing habit.” (Participant 16, Male, Urban school)

Highly Engaging

The students agreed that the app was easy to navigate. This was primarily due to its simple and intuitive interface, which allows users to quickly access key features without confusion or difficulty. The layout was clean, and the icons were clear, making it straightforward to perform tasks such as setting reminders or tracking progress. Moreover, there are features within the app that motivate them to use it, such as the games and habit tracker. These features made them want to use the app until their names are displayed in the leaderboard.

“I feel comfortable using this app because the user interface is very direct, and it can help people who may not know anything about it, but they can easily understand how to use it.” (Participant 21, Female, Rural school)

“For me, the games and the habit tracker are what motivate me to use this app. In the game, if we get a high score, we enter the leaderboard, so it makes me want to keep playing until I get a perfect score so I can appear on the leaderboard. Getting into the top 10 is good enough for me.” (Participant 16, Male, Urban school)

Feasible

The children agreed that every activity in the app they were involved in was reasonable and had its benefits. They felt the app was feasible to use, the features were clear and not confusing, and they could also learn English while using it.

“For me, this app is easy to open, use, download, and navigate. It doesn't take a lot of sensitive personal information; it only takes information related to your teeth.” (Participant 22, Female, Rural school)

“I think the features are clean, clear and not confusing. First, the app is in English, so everyone can also learn English. Many people can use this app. People of all backgrounds can use it. All the features are easy to use, just click and tick.” (Participant 19, Male, Urban school)

“All the terms used in the app are easy to understand…” (Participant 9, Female, Rural school)

Lack of Time

Some students who admitted to having tight schedules and limited time to report their oral health activity tasks in the app showed a lack of interest in using the app, including those who were less interested in reading, as the app provided ample information on oral health.

“The only thing is with the tracking; I feel like we need to commit ourselves to reporting and tick things off in it. Because if we’re too busy, we might forget to tick...” (Participant 18, Male, Urban school)

“I think maybe for people who already have a well-organised schedule, they might not need to use this app. Similarly, for those who don’t like to read, like me.” (Participant 16, Male, Urban school)

Ethicality

Ethicality refers to the extent to which the MySmile app aligns with users’ values or norms. In this domain, only one theme emerged, i.e., cultural or personal value alignment.

Cultural or Personal Value Alignment

All participants agreed that this app did not conflict with their personal values and societal norms. All the content within the app was relevant to teenagers and did not contradict their personal ethics or societal values.

“Frankly speaking, I think the MySmile app is reasonable in helping the community, especially teenagers. No ethical issues. It serves as a reminder, provides information at your fingertips regarding oral health, and, at the same time, offers entertainment through games.” (Participant 19, Male, Urban school)

“Are the contents contradictory to societal norms or my principles? I don’t think so. I feel that this app is very useful both at an individual level and for society. It’s beneficial to all of them.” (Participant 20, Male, Urban school)

Intervention coherence

Intervention coherence relates to how well users understand the purpose of the MySmile app. Two themes emerged, i.e., understanding and clarity, and perceived relevance and coherence.

Understanding and Clarity

The students demonstrated a clear understanding of the purpose and functions of the MySmile app. They were able to describe how the app contributed to their oral health awareness and provided useful guidance in performing proper oral hygiene practices.

"It teaches us about proper dental care. From not knowing, we come to understand how to take care of our teeth." (Participant 4, Male, Urban school)

Some users reflected that the app’s features, such as the quizzes and sugar tracking, can provide them with feedback that encouraged behavioural change. They interpreted the app as a tool that made them more aware of their habits and prompted them to act on that awareness.

"After two weeks, I consumed a lot of sugar, and then I looked at my report in the Habit tracker. so I tried to reduce it." (Participant 6, Female, Rural school)

Others commented on how the app's layout and structured sections helped them understand what the app was asking them to do and how to navigate it.

"When I first downloaded it, I thought it would be difficult to use this app. But once I opened it, all the instructions were there so I understood exactly what to do." (Participant 6, Female, Rural school)

Perceived Relevance and Coherence

The students agreed that the app made sense to their lives, routines, and needs as adolescents. They saw the app as relevant, both in content and timing, filling gaps in OHE that were not typically addressed elsewhere.

"I think this app is suitable for teenagers because we are not really exposed to dental care. It's mostly about skincare, but we don’t really know how to take care of their teeth." (Participant 10, Female, Rural school)

The app was also seen as fitting naturally into daily routines. Users noted that the brushing tracker and reminders aligned well with them when they brushed their teeth, helping to reinforce existing habits without being disruptive.

"The content in this app really fits our lifestyles. We can tick off our brushing habit and sugar intake at the habit tracking feature, and even better, it provides a report in the form of a graph.” (Participant 10, Female, Rural school)

Some mentioned that the app felt personally meaningful because it offered a sense of control and insight over aspects of their oral health they previously ignored.

"I like this app because it makes it easier for me to track my dental health and sugar intake, which I never really paid attention to before." (Participant 4, Male, Urban school)

Moreover, these insights show that users not only recognised what the app was intended to do but also grasped how it worked to achieve its health goals. This suggests that the app was perceived as a coherent, meaningful intervention capable of influencing daily health practices in a purposeful way.

"I like this app because it makes it easy for me to track my oral health and sugar intake. I also like the games provided; they are very enjoyable. The app is well coherent with my lifestyles." (Participant 9, Female, Rural school)

Opportunity cost

Opportunity cost relates to what users have to give up when engaging with the app. In this domain, only one theme emerged, i.e., no perceived trade-off.

No Perceived Trade-Off

Majority of the students felt that they hadn’t sacrificed any of their valuable time or other daily activities to use the MySmile app. The time required to engage with the app was perceived to be short and flexible, allowing them to fit it easily into their day routine without needing to give up anything. They also agreed that using this app did not take their time away from other more important tasks. Some even felt that using the app added value without compromising on schoolwork, rest, or leisure. Hence, they did not perceive the presence of any opportunity cost associated with using the app.

“For me, I don’t have to give up anything precious because I use this app. It doesn't require much time because all I need to do is open the app and click through it. And somehow, as teenagers, we can’t seem to be away from our phones. So, I feel I can easily use this app whenever I have some free time.” (Participant 8, Female, Rural school)

“For me, the MySmile app doesn't take my time at all. I can still do anything I like, and I feel that it doesn't interfere with my daily routine.” (Participant 6, Female, Rural school)

Another important aspect of the trade-off is that the students felt that the app did not impose any financial cost on them. As the app was free to use, they could benefit from its features without worrying about hidden fees or subscription costs.

“There’s no issue because the app’s download size isn’t that large, and it doesn’t take up much space. Plus, the app is free, and we can get a lot of benefits from it.” (Participant 4, Male, Urban school)

Perceived effectiveness

Perceived effectiveness relates to the extent to which users believe the intervention achieves its intended goals. In this domain, two themes emerged, i.e., improve oral hygiene practice and improve knowledge of oral health.

Improve Oral Hygiene Practice

The students unanimously agreed that the MySmile app helped them to improve their oral hygiene practice, particularly in terms of brushing frequency and techniques. The majority described that the app’s features, such as Reminders and the Habit tracker, had supported them in making positive behavioural changes over a relatively short period.

"I used to always forget to brush my teeth, especially at night. Now, this app reminds me to brush my teeth before I go to bed. So I think this app is great." (Participant 5, Male, Rural School)

They also agreed that the Habit tracker function alerted them when their sugar intake exceeded the recommended limit, which prompted them to make a healthier choice.

“At first, I used to mark that I had sugar 5-6 times a day. So, I would tick off the habit tracker. Then, my phone vibrated like it was angry because I had already reached the maximum sugar intake for the day. From that point on, I tried my best to follow the app's suggestion to have sugar less than 4 times sugars per day.” (Participant 2, Male, Urban school)

Improve Knowledge on Oral Health

The majority believed that the app was effective in helping them achieve their goals and gain knowledge about oral health. They also felt that the app enhanced their understanding of toothbrushing techniques, flossing, sugar intake limits, smoking, and common dental problems. The educational content of the app, including videos, quizzes, games, and the Knowledge Hub, greatly contributed to their learning process and knowledge acquisition.

“In my opinion, this app is truly effective if we focus on using it. Not only is it great for providing knowledge, but it also helps discipline me. For example, with my sweet food intake, I didn’t know the maximum limit before. After using the app, I now know the limit. The quizzes and games really help.” (Participant 2, Male, Urban school)

“I learned how to brush my teeth properly by watching the video in the Knowledge Hub, because before this, I just brushed randomly and didn’t even know the correct techniques. I also know how to floss properly. Also, good info on smoking etc..” (Participant 20, Male, Urban school)

Self-efficacy

Self-efficacy relates to their confidence in their ability to carry out the behaviours encouraged by the app. In this domain, only one theme emerged, i.e., perceived control over health behaviours.

Perceived Control Over Health Behaviours

Most participants felt empowered and believed they could reduce their sugar intake after using the app and receiving consistent reminders or feedback. Additionally, some participants reported gaining the ability and confidence to change their brushing habits after using the app, which contributed to improved self-efficacy.

“The feature I like the most is the Habit tracker. Through this feature, I believe I can control my sugar intake and become more diligent about brushing my teeth. I’m confident I will be able to control my sugar intake and consistently brush my teeth, especially before bed.” (Participant 2, Male, Urban school)

“I saw a video on how to brush teeth properly using the circular motion technique. Before this, I brushed my teeth randomly, but when I started using this app, I followed the proper brushing techniques. After a month, I’m now confident in brushing my teeth with correct technique.” (Participant 3, Male, Urban school)

“Overall, I can say that my sugar intake has decreased, and I’m confident that I can reduce it further..” (Participant 21, Female, Rural school)

## Discussion

This study explored the acceptability of the MySmile app among Malaysian secondary school students using the TFA [9]. The findings demonstrated that the app was well-received across all seven TFA domains, suggesting that the MySmile app holds promise as a digital health intervention to support oral hygiene among adolescents. This discussion elaborates on how each domain of the TFA contributed to shaping the app’s perceived acceptability, drawing directly from the voices of participants and supported by existing literature.

In terms of affective attitude, most students expressed positive emotions when using the app, describing it as fun, and informative. These feelings were driven by the appealing design, gamified features, and relatable content, which enhanced motivation to engage with oral health information. As the students explained, the app helped in making dental knowledge easier to understand. This aligns with findings in adolescent mHealth research, where emotionally positive user experiences enhance uptake and engagement [17]. However, a minority expressed negative emotions. They stated their concerns about the app's legitimacy for not being officially published on mainstream app stores like Google Play or the App Store, a general lack of familiarity with such digital tools, or technical issues preventing login. This mirrors challenges observed in mobile health implementations, where user trust in the platform and initial onboarding friction can hinder adoption, even for a well-designed app. This aligns with a finding from a study by Shirmohammadi, Razeghi, Shamshiri and Mohebbi [18], who found that practical barriers and user hesitations could limit the reach of an oral health app, highlighting the gap between potential and actual use in real-world settings. Despite this, several participants showed enthusiasm for using the app and proposed enhancements, including a bilingual version and the addition of a reward system to maintain engagement. These suggestions reflect the desire for culturally and linguistically responsive content and indicate that while the affective experience was largely positive, fine-tuning is needed for full emotional alignment [19].

Regarding the burden domain, participants described the app as effortless to use. They found that the MySmile app required minimal time and could be easily integrated into daily routines. This was attributed to the intuitive layout, simple navigation, and features like reminders and the Habit tracker that reinforced routine behaviours. These findings are consistent with evidence that digital health tools perceived as low burden are more likely to be used consistently [20]. Furthermore, participants found the app engaging and enjoyable due to its game-based structure, which motivated some to compete on the leaderboard. This element of gamification added a sense of reward and reinforced user interest [21]. However, some participants, especially those with busy schedules or less interest in reading, felt that time constraints limited their engagement. They suggested that while the app was feasible, its effectiveness could be reduced for users who lack intrinsic motivation or time management skills. This highlights the importance of designing mHealth tools that accommodate varying user needs and lifestyles [22].

In the domain of ethicality, all participants agreed that the app aligned with their personal values and cultural norms. They appreciated that the app did not require invasive data or personal sharing, which contributed to a sense of safety and trust. The content was viewed as socially appropriate and age-relevant, with participants affirming its value to teenagers and society as a whole. This acceptance suggests that the app met a critical ethical threshold for acceptability by aligning with users’ expectations of respectful and culturally sensitive health communication [9].

Under intervention coherence, participants demonstrated a clear understanding of the app’s purpose. They recognised that features such as quizzes, videos, brushing trackers, and sugar intake logs were intended to improve oral health behaviours. Several participants noted that the structured layout made it easy to follow, and many interpreted the app as a tool for both self-monitoring and behavioural reflection. These interpretations reflect high levels of coherence between what the app offers and how users understood and used it. Importantly, students also discussed how the app filled a gap in OHE, particularly for teenagers who are often overlooked in traditional programmes. The feedback suggests that the MySmile app was perceived not just as informative, but as meaningful and relevant to their lived experiences. This strong perceived coherence, where the educational content was effectively complemented by interactive elements such as games and reminders, created a consistent and meaningful user experience, enhancing its overall acceptability [23]. 

The opportunity cost of using the app was considered negligible. Participants indicated that they did not need to sacrifice time, academic performance, or social engagement to use the app. The app’s short usage time, flexible schedule, and low resource requirements made it easy to integrate into daily life. Furthermore, participants appreciated that the app was free and accessible on devices with limited storage. These factors align with prior research indicating that low-cost, low-barrier interventions are essential for uptake in adolescent populations [24]. The lack of perceived trade-offs further strengthens the case for integrating the MySmile app into existing health promotion strategies in schools.

In terms of perceived effectiveness, students reported behavioural and cognitive changes resulting from the app’s use. Many participants reported improvement in brushing habits, reduced sugar intake, and better awareness of oral hygiene practices. The habit tracker, reminder system, and educational videos were key drivers of these outcomes [25]. Students also expressed appreciation for learning new information, such as proper brushing techniques and sugar limits, which they had not previously been exposed to in formal education. These perceived outcomes support the app’s potential to meet its intended goals and contribute meaningfully to oral health literacy development [26]. Similar results have been reported in previous mHealth interventions targeting adolescent behaviour change [27].

Finally, the theme of self-efficacy emerged strongly. Participants expressed increased confidence in their ability to manage oral hygiene independently. They believed that they could sustain healthier behaviours such as reducing sugar intake and brushing effectively without external supervision. As some participants stated, the app helped them become more disciplined and enabled them to take control over their oral health habits. This sense of empowerment is crucial in adolescent health promotion, where building autonomy and self-regulation is key to long-term behaviour change [28,29]. The design of the app appears to have supported the development of these skills by making health tasks manageable and personally relevant.

While the findings demonstrate high acceptability of the MySmile app within the sampled urban and rural schools in the Kinta district, the transferability of these results to other regions in Malaysia should be considered with caution. Differences in digital access, socioeconomic background, and linguistic preferences may influence how adolescents engage with mHealth interventions elsewhere [30]. For example, schools in remote or under-resourced areas may experience challenges such as limited smartphone ownership or inconsistent internet connectivity. Future research should therefore examine the acceptability of the app across a broader range of school types, including vernacular, religious, and boarding schools, to enhance generalisability and ensure equitable implementation at the national level.

Digital oral health tools like the MySmile app can play a transformative role in adolescent oral health promotion when they are engaging, accessible, and aligned with users’ values and lifestyles. Grounded in user experience and theory, the app has the potential to be scaled up as a practical, acceptable solution for improving oral health knowledge, attitudes and behaviours in school settings.

The integration of the MySmile app into existing national oral health initiatives, such as the School Dental Service in Malaysia and other nations in the region, offers a strategic opportunity to enhance reach and relevance. Currently, the School Dental Service relies heavily on periodic dental visits and traditional educational materials, which may not sustain long-term behavioural change. Incorporating the MySmile app as a digital adjunct could modernise and personalise oral health promotion for adolescents, particularly in settings where dental personnel resources are limited. The app’s alignment with school-based health strategies and its demonstrated acceptability suggest it could be scaled nationally as part of a blended learning model in OHE. Collaboration between app developers, dental public health authorities, and the Ministry of Education would be essential for successful implementation.

Taken together, these findings offer a comprehensive understanding of how adolescents experience, interpret, and apply a digital oral health intervention in their daily lives. They highlight the critical role of user-centred design, cultural sensitivity, and behavioural relevance in fostering acceptability. Overall, the positive attributes generated from the themes were positive emotions, enthusiasm towards participation, cultural value alignment, no afford required, highly engaging, feasible, no perceived trade off, perceived control over behaviours, clarity, perceived relevance and coherence, and improved oral hygiene practice and knowledge. Only 2 negative attributes generated from the themes, i.e., negative emotions, and lack of time. In this study, the MySmile app shows strong potential for broader implementation, provided that technical improvements and content expansions are considered. Based on the findings, the apps can be improved further by incorporating a bilingual version (Malay and English) for users, and a reward-based system to maintain engagement across diverse adolescent groups. Further research may also explore long-term adherence, family involvement, and integration with school-based oral health policies. Importantly, the use of the TFA allowed for a structured, multidimensional understanding of acceptability, which is critical for refining interventions before large-scale implementation.

This study has several limitations. First, the findings were based on FGD with a purposively selected sample from two schools, which may limit the generalisability of the results to other populations or cultures. Therefore, the acceptability of this app was limited to the sample population within their sociocultural environment. Second, participants may have been influenced by social desirability bias, given the group setting and the presence of the facilitator with a professional background in oral health. Although steps were taken to foster open discussion, such bias may exist to some extent. Third, the study was conducted in the Kinta district where the study population did not represent all ethnic groups. Therefore, the study findings may represent the sample population only. Lastly, the study focused solely on short-term user perceptions of the app’s acceptability. Long-term usage patterns, retention, and acceptability were not assessed in this study.

## Conclusions

This qualitative study demonstrated that the MySmile app was well received by the students across all seven Theoretical Framework of Acceptability domains. Students reported strong emotional engagement, minimal burden, and a clear understanding of the app’s purpose. The app was aligned with their values and perceived as highly relevant to their daily routines and oral health needs.

The app was perceived as effective in promoting brushing and flossing habits, reducing sugar intake, enhancing oral health knowledge, and increasing confidence in sustaining these behaviours among the target group. While most experiences were positive, minor usability concerns such as unclear navigation and limited personalisation options were raised. These findings highlight the importance of incorporating user feedback to further optimise the design and delivery of mHealth tools. Future efforts should consider expanding the app’s features to support diverse learning styles and linguistic needs and evaluating its long-term impact on oral health behaviours.

## Figures and Tables

**Figure 2:**
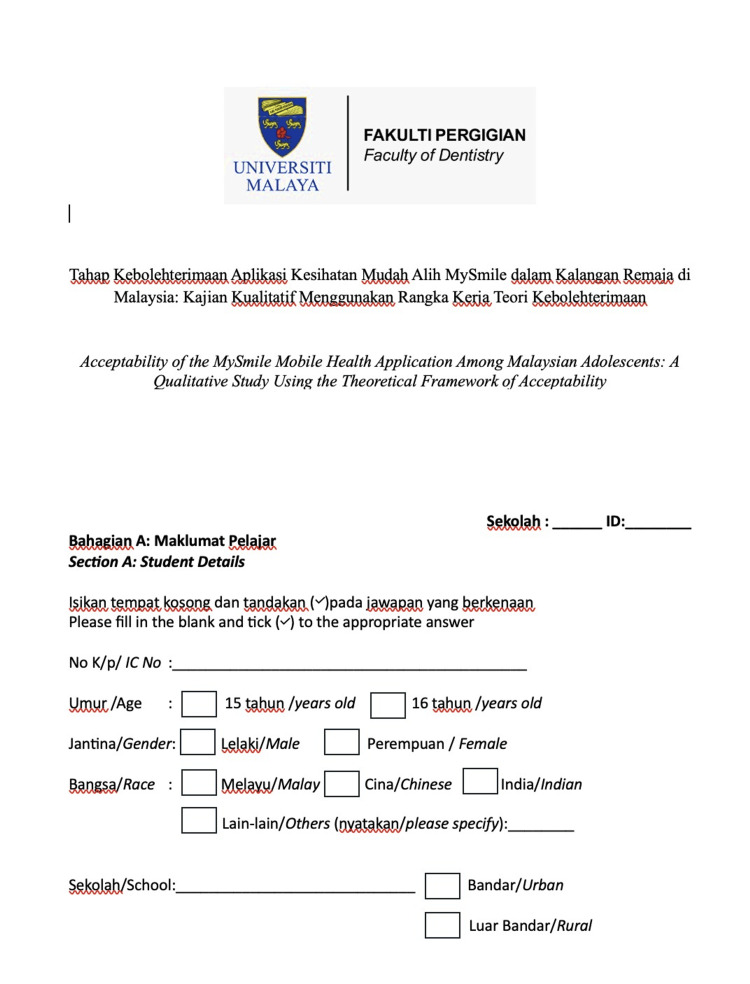
The sociodemographic form.

## Appendices

**Table 2:** Semi-structured topic guide of the Focus Group Discussion

Construct	Leading question	Probing question
Affective Attitude	How do you feel about MySmile App / How would you describe your experience using the MySmile app? Bagaimana perasaan anda tentang Aplikasi MySmile? / Bagaimana anda menggambarkan pengalaman anda menggunakan aplikasi MySmile?	Do you feel the content and features of the app are relevant to your needs? Why or why not? How well does the app fit into your daily routine or lifestyle? Do you feel comfortable using the app regularly? Why or why not?
Burden	How would you describe the level of effort needed to participate in this intervention Bagaimana anda menggambarkan tahap usaha yang diperlukan untuk menyertai intervensi ini?	What tasks or activities in the MySmile app do you find most demanding? Do you feel the effort required to use MySmile app is reasonable? Why or why not? What motivates you to invest the effort needed to use MySmile app?
Ethically	How well does this app align with your personal values or beliefs? Sejauh manakah aplikasi ini sejajar dengan nilai atau kepercayaan peribadi anda?	Are there any aspects of the intervention that feel consistent or inconsistent with your cultural or ethical values? Does the MySmile app feel meaningful or personally relevant to you? If so, how? Does MySmile app inspire or motivate you to use it? Why or why not?
Intervention coherence	Can you explain in your own words how the MySmile app works? Boleh anda terangkan bagaimana MySmile app berfungsi?	Are there any features of the app that you find unclear or confusing? How confident are you in navigating the different sections of the app? Do you understand how to use all the features provided by the app? If not, which ones? Do you feel the instructions or guides within the app are clear and easy to follow?
Opportunity cost	Do you feel you have to give up anything significant to use the MySmile app? If so, can you tell me? Adakah anda rasa anda perlu mengorbankan sesuatu yang penting untuk menggunakan aplikasi MySmile? Jika ya, bolehkah anda beritahu saya?	How much time do you feel you need to dedicate to using the MySmile app effectively? Are there any financial costs (e.g.,data usage) associated with using the app that you find burdensome? Does using the app take away time or energy that you would prefer to spend on other activities?
Effectiveness	How effective do you think the MySmile app is at achieving its intended purpose? Sejauh manakah keberkesanan aplikasi MySmile dalam mencapai tujuan yang dimaksudkan, menurut pandangan anda?	What specific improvements or benefits have you experienced from using the app? Do you think the app addresses your oral health needs effectively? Why or why not? How confident are you that the app will help you achieve your oral health goals? Have you noticed any measurable changes in your oral health or habits since using the app? Are there any limitations or challenges that prevent the app from achieving its purpose for you?
Self-efficacy	How confident do you feel in your ability to perform the recommended oral health behaviours after using the MySmile app? Sejauh manakah keyakinan anda terhadap keupayaan anda untuk melaksanakan tingkah laku kesihatan mulut yang disarankan selepas menggunakan aplikasi MySmile?	What specific skills or behaviours have you learned or improved through using the app? Can you confidently apply the knowledge and techniques from the app to your daily routine? Are there any behaviours recommended by the app that you find difficult to perform? If so, what makes them challenging? Do you think you can continue performing the recommended behaviours consistently in the future?
